# Understanding the Preferences of Genetic Tools and Extension Services for the Northern Australia Beef Industry

**DOI:** 10.3390/ani16010132

**Published:** 2026-01-02

**Authors:** Patricia Menchon, Amy Cosby, Dave L. Swain, Jaime K. Manning

**Affiliations:** 1 Institute for Future Farming Systems, School of Health, Medical and Applied Sciences, CQUniversity Australia, Rockhampton, QLD 4701, Australia; a.cosby@cqu.edu.au (A.C.); dave.swain@terracipher.com (D.L.S.); j.k.manning@cqu.edu.au (J.K.M.); 2 Centre for Research in Equity and Advancement of Teaching and Education (CREATE), CQUniversity Australia, Rockhampton, QLD 4701, Australia; 3 TerraCipher, 337 Laurel Bank Rd., Alton Downs, QLD 4702, Australia

**Keywords:** genetic, genomic, beef, adoption, stakeholder, extension, producer

## Abstract

This study aims to understand the motivations, barriers, and preferences of northern Australian beef producers to adopt genetic tools through the views of different stakeholders. Using qualitative research with a single-case study approach, 15 semi-structured interviews were conducted to collect data which were then thematically analysed. Motivating factors to adopt genetic tools were the usefulness of genetic information, the productivity gains, and the profit of the beef enterprise. Barriers to adopting genetic tools included individual factors such as the lack of understanding, limited education, and the attitude of producers or contextual factors such as geographical location and size of production systems. This knowledge will support the development of future extension interventions to promote the use of genetic tools. To effectively promote the use of genetic tools in northern Australian beef production, extension programs should consider both the environmental and geographical context as well as the attitudes and beliefs of local beef producers. This study could present limitations related to sample bias. Future research should include more representative samples and mixed-methods approaches, supplemented by analyses of case studies to validate the reported perceptions.

## Simple Summary

Beef producers in northern Australia are slow to adopt genetic tools that could improve cattle performance. We interviewed 15 people from the beef industry to find out why. Producers see benefits like better productivity and profits, but face challenges such as limited knowledge and the difficulty of collecting data in remote areas. Tools like polled gene testing are popular, while others, like estimated breeding values, are less understood. To help adoption, producers prefer practical, face-to-face learning in small groups led by experienced peers. These insights will guide programs that make genetic tools easier to use and more valuable for producers.

## 1. Introduction

Beef production is the main agricultural activity in northern Australia, making it a major contributor to the region’s economic, social, and environmental factors [1]. Northern Australia beef enterprises have lower stocking rates than in southern Australia due to the lower carrying capacity of mostly native pastures; however, they encompass approximately 55% of the region’s land area and contribute 70% of all Australian beef cattle [1,2]. The climatic variability, including seasonal rainfall, as well as occurrences of extreme climatic events (e.g., floods and droughts) reinforce the need to consider genetic improvement, grazing management strategies and stocking rate when producers are making decisions [3,4]. Although there are opportunities for productivity improvement through feed innovations, genetic selection and technology adoption emerge consistently as key to improving the northern Australian beef industry [1,3,4,5,6].

The adoption of technology in agriculture is a broad concept that includes its development, dissemination, and implementation by the end-user on farms. The main factors that affect the adoption of technology through behavioural intentions are individual beliefs such as performance expectancy, effort expectancy, social influence, and also habits and facilitating conditions [7,8]. The adoption of technology is presented as a dynamic process [9,10] that demands a holistic vision [11] which pursues the general objective of increasing productivity, efficiency, and profitability. Pannell and Vanclay [9] reinforce the idea that it is a complex process and summarise that adoption processes are affected by social networks and local producer organisations, proximity to another producer adopting technologies, physical distance from the information source, trust, and the develop of extension programs from both government and private sector.

Frameworks and models have been developed to explain the adoption of new technologies and factors that may influence user acceptance [12]. According to Chilcott, Ash [1], despite the efforts made to encourage the adoption of best management practices, it continues to be a key issue for beef industry. This has similarly been reported by Sun, Hyland, and Bosch [11] and Jakku, Farbotko [6].

The reviewed literature shows limited use of theoretical frameworks and models in research on genetic tool adoption in beef cattle. These theories are vital in extension research, as they help explain technology adoption. In research on genetic selection in dairy cattle, Ooi, Stevenson [13] utilised the Theory of Planned Behaviour (TPB) and found that producers had a wide range of opinions and attitudes about fertility and genetics in the process of sire selection. The TPB [14] is a theoretical framework which places special emphasis on attitudes, intentions, and perceived significance. The theory has also been utilised in the dairy and sheep sectors in southern Australia [15,16], and it found that beliefs, behavioural attitudes, and perceived behavioural control should be considered in initiatives that seek to promote change in practice. Adding social influences, the Unified Theory of Acceptance and Use of Technology (UTAUT) [7,17] considers performance expectancy, effort expectancy, social influence, and facilitating conditions as the main factors that affect adoption, but with demographics such as gender, age, experience, and voluntariness of use. Research to increase knowledge of the adoption process of technology related to the genetic improvement of cattle based on theoretical frameworks is still incipient with further exploration necessary with a focus on the northern Australia beef industry.

This study aimed to understand the motivations, barriers, and preferences of northern Australian beef producers to adopt genetic tools through the views of different stakeholders. The research questions (RQ) driving this research are RQ1: How do producers make decisions to adopt genetic tools and why do some producers adopt them while others do not? RQ2: What are the preferred genetic tools and why? and finally, RQ3: What are the sources of information of beef producers for content related to genetic improvement?

## 2. Materials and Methods

This study was based on qualitative research [18] with a holistic single-case study approach [19,20,21] using interviews to collect data [22]. The single-case study under revelatory rationale [21] was selected to obtain further in-depth understanding of factors influencing the adoption of genetic tools in the northern Australia beef industry. To have a broad representation of all the people involved in the northern Australia beef industry, semi-structured interviews were conducted with representatives of beef cattle breed societies, extension officers, advisors, producers (stud and commercial) and stock agents. Consequently, this study takes a constructionism approach [23] because it is intended to generate an understanding of motivations, barriers, and preferences of northern Australian beef producers to adopt genetic tools considering the context through data collection and analysis.

Incorporating a theoretical framework in extension research allows us to understand the cognitive factors (expectations, efforts and social influences) that affect the adoption process, allowing these to be incorporated into an extension program [8]. The theoretical multi-level framework UTAUT/UTAUT2 [7] is considered in this study. This extension of UATUA [17] is selected because it considers not only social factors but also the context of the end user consumer that explains the acceptance and use of technology.

This research was approved by CQUniversity Australia Human Research Ethics Committee (approval number 24458). The interview process and how the thematic analysis [24] was developed and will be discussed below.

### 2.1. Semi-Structured Interviews

Semi-structured interviews were conducted in person or online with stakeholders. Recruitment and interviews were conducted between December 2023 and February 2024. Participant recruitment was conducted via social media and email invitations. The objective of each interview was to find out stakeholders’ knowledge, opinions, and experiences of the use of genetic tools and their dissemination among producers in northern Australia. Through the exploratory interviews, detailed answers were sought to understand the motivations and barriers for beef producers in northern Australia to adopt genetic tools. The divergences in perspectives and roles among the diversity of stakeholders supposes the plurality of types of collaborations that can be carried out [25,26,27,28]. Therefore, a semi-structured and open questionnaire was used to answer the research questions from this study according to their roles. The questionnaire was discussed and developed with researchers from the CQUniversity Agri-Tech Education and Extension Research team and was validated with two expert researchers related to beef production and animal genetic improvement. Questions focused on perceived barriers and motivations for the adoption of genetic tools, preferred delivery methods used in extension activities, and communication strategies with producers.

Purposive sampling was used to conduct detailed interviews with interviewees. This allowed for the inclusion of participants with diverse levels of adoption or understanding of genetic tools, to reduce potential bias. Initially, invited participants received a clear explanation of the research purpose via a Participant Information Sheet outlining the aim, interview process, and personal data use. After obtaining verbal consent from the interviewee, a guide consisting of 8 questions related to the adoption and use of genetic tools was used for the interview. The interviews were conducted in person, by Zoom meetings or by phone calls by two researchers. Fifteen interviews were conducted as this was the point where no new themes emerged and the dataset was considered saturated [22].

### 2.2. Participants

The participants were characterised by developing different roles within the northern Australia beef industry. Due to the overlapping roles for held by individual interview participants, only their main role mentioned and counted for description purpose. The number of participants was 15 stakeholders (ID1 to 15), of which 73% were males and 27% were females. The roles were commercial beef producer (*n* = 2), stud beef producer (*n* = 6), extension agent (*n* = 1), genetic researcher (*n* = 1), stock agent (*n* = 3), business advisor (*n* =1), and staff member of breed society (*n* = 1). The age range of the participants was between 21 and 70 years of age.

### 2.3. Data Analysis

**Figure 1:**
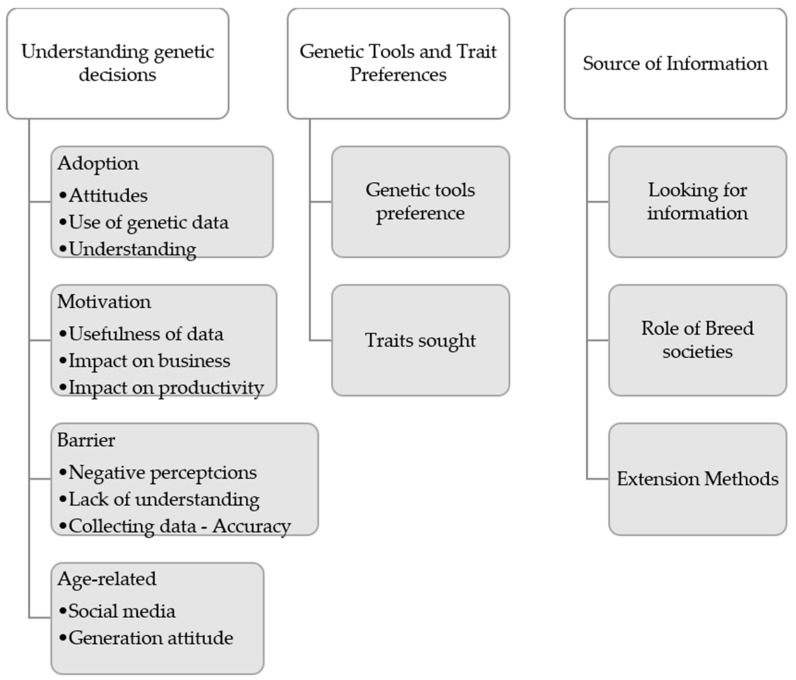
Themes identified through interviews with northern Australian beef industry stakeholders.

To analyse the data that were collected, the procedure of thematic analysis proposed by Braun and Clarke [24] was followed. The interviews were recorded and transcribed using Otter Ai [29]. The analysis approach is inductive, where the codes emerged from the responses of interviews. After defining the codes and subcodes, connections were established with the constructs of the UTAUT/UTAUT2 model. These connections allow the points of convergence between the inductive findings and the theory to be identified. The analysis of codes and themes was carried out using the NVivo 1.7.1 (1534) tool software [30]. The themes (codes) that emerged through interviews and were related to the research questions were classified and grouped for analysis (Figure 1). To reduce the risk of overgeneralization, the interview data were analysed by identifying stakeholder roles before aggregation. This allowed researchers to determine whether emerging themes varied systematically by role or if there was convergence of themes. According to Creswell and Poth [18], there are several ways to validate qualitative data analysis. When it is not possible to use quantitative data or other external sources in an exploratory study, a helpful option is to use a stepped peer-review process, where emerging codes and analyses are reviewed at different stages. This step-by-step approach helps researchers gradually improve the coding structure and work toward a consistent and shared interpretation, as was performed in this study. It is also important to clearly state the study’s limitations and any potential biases that could affect how the findings are interpreted.

## 3. Results and Discussion

Themes and sub-themes are presented in Figure 1 and further discussed in the following sections.

### 3.1. Understanding Genetic Decisions

To answer RQ1: How do producers make decisions to adopt genetic tools? And why do some producers adopt them while others do not? The state of adoption, motivations, and barriers that stakeholders face in the decision-making process to use genetic tools were analysed.

#### 3.1.1. Current Level of Adoption

Interview results suggest that there is low adoption of genetic tools in northern Australia, although there is a change towards an increase in the intention to adopt. In line with what was found by Morris and Holloway [31], participants reflected on different approaches and contextual realities that characterise the use (or not) of genetic tools. When asked about the use of genetic tools, some participants remarked on the low level of adoption saying that “you can definitely see commercial fellas [producers] still have that idea that they don’t” (ID11) use genetic information in their herds, with another stating that “adoption is going to take time on the north” (ID2) and “there’s not that much genetics out there, data out there” (ID14). A common view amongst participants was that there has been a slow change in the adoption trend. This is supported by quotes such as “progress is something that we must take note of”, “EBVs [estimated breeding values] are coming in. I sort of used to roll my eyes a bit and then I’ve realised that no, that they do have their place” (ID10), and “I think it’s slowly like some [producers] are definitely using it [genetic information] more than others” (ID5). The regional characteristics that determine the beef production system seem to affect adoption as well, “because of where we are in [location] Queensland and obviously a lot of bulls are ready to go to North Queensland … that focus on EBVs probably has been limited” (ID12), and another stakeholder said “it’s gathering momentum, and especially with being able to take samples and you know, quickly get some data on an animal without having to measure any raw data on that animal” (ID2).

#### 3.1.2. Motivations for Adoption of Genetic Tools

Knowing the drivers for the use of genetic data is crucial to designing effective adoption strategies. Stakeholders were asked about what motivates them to use genetic tools in their herds, with three themes emerging: the usefulness of genetic information, the productivity and the business impacts of beef enterprise.

The usefulness of the genetic data was associated with the choice of traits desired in the offspring that cannot be selected through the phenotype of the parents. This is particularly important for characteristics such as birth weight, because it is one of the factors that defines the survival and health of calves and cow mortality. Recording the birth weight of calves would allow genetic evaluation that would compare animals and identify animals whose birth weight genetics differ from their post-birth growth [32]. The usefulness of genetic information was highlighted by “you can’t tell the birth weight of a bull when you’re looking at him, but you can when you’re looking at the data” (ID10). Additionally, seeing the results of changes in the genetic traits of cattle through genetic selection was another factor. For instance, one participant said, “you can see the improvement in your herd” (ID3), and another said “if you’re selecting genetic [ally] superior animals, then you’re increasing the frequency of favorable genes in the herd” (ID1). However, another perceived the usefulness of genetic tools as having access to data per se, one participant indicated that “the more information I’ve got, the better” (ID6), and also stated that “some are probably wanting more information on their herd, so they can then give that information to customers or clients”.

Improved productivity of the beef production system was another motivation and, in some cases a necessary condition for the uptake of genetic tools in northern Australia with one participating saying “I don’t think they would use it unless it did … improve productivity. I think that would be the key driver towards it … [and] I think productivity would be the key driver for them wanting to use it that [they] have to see a benefit for their business” (ID7). Productivity must be understood and analysed through the collection of data in a systematic and practical way [4], as this would make it possible to understand opportunities for improvement. However, the increase in productivity will also need to be cost-effective to achieve improvement in profitability, which means the returns from the additional productivity will need to higher than the cost of use [1]. Nevertheless, the majority of the participants suggested that improvement of the business is a significant driver for adopting the use of genetic tools.

The beef production chain is a complex industry with diverse stakeholders with overlapping economic interests. The genetic progress of the entire beef industry in a particular productive region is determined by the genetic selection of traits carried out by stud producers [33]. This is further influenced by bulls transferred through purchase and sale to commercial herds. General expressions such as “I suppose at the end of the day, what it’s all about, it comes back to money at the end of the day, no matter what you do” [and] “how quickly you can get dollars in the bank” (ID10) with another stating, “bottom line, profitability, be the key” (ID4), which affirms the importance of the economic perspective. The improvement in the price of the genetically superior animal achieved by stud producers could lead to a higher financial cost experienced by commercial herds purchasing bulls. Consequently, the motivation of the use of genetic tools from an economic perspective focuses on the achievable profit over the assumed expenses. This demonstrates the differences in terms of motivations to adopt genetic tools. For instance, when comparing EBV’s on 200 and 400 day weight traits, one commercial producer said “there’s something going on there that could be quite valuable for you when it comes back to calves bred by that animal …. The money’s there, because he’s [the calf] still growing and making you money” (ID10), and a stud producer said “the money they (commercial producer) spend … I think it’s going to be to the stage where they won’t buy bulls, unless they’ve got the paperwork […] so basically, we need that information to supply to our commercial breeders” (ID13).

#### 3.1.3. Barriers to Adoption of Genetic Tools

There are a variety of barriers that limit the adoption of genetic tools including the lack of understanding, available education, and attitude of producers in northern Australia. One participant said “mainly education, I think is a key area that you can be worked on in especially northern Australia” (ID2), and this was reiterated by “more education, we definitely believe that would be helpful for everybody” (ID6). This is important because the lack of understanding regarding genetic tools, whether one’s own or the perception about other producers are associated with the absence of reasons to change decision-making [34]. For some producers “when they don’t understand that they don’t see value in it, you know, they don’t like change” (ID2), or in reference to specific traits such as fertility and growth, “if they don’t understand, there may be some fear that they will lose something by it” (ID7) demonstrates the attitude differences of producers to genetic tools. The effect of education on technology adoption is heterogeneous according to previous literature reviews [35,36]. For instance, Montes de Oca Munguia and Llewellyn [36] claims that the level of education has a variable effect on adoption according to the type of innovation with Menchon et al. [35] finding that the level of education of the producer has different effects on the adoption of genetic improvement tools. However, education is a recurring barrier to adoption that was identified in Australia and has motivated the implementation of practices that involves educational material, awareness activities, skills training and participatory projects [4,37,38]. Commercial producers also recognise their lack of understanding and need to continue learning about genetic tools. This is aligned with the opinion of stock agents who recognise that it is not easy for beef producers to apply genetic concepts and that it is difficult to implement them, although they are making efforts to do so. Even more so, the stock agents who act as intermediaries in the purchase and sale of animals between stud, commercial farms and the beef industry, mention that there are many agents who do not believe in genetic tools, but that they are always responsive to the demands of the client. Therefore, the need for education related to genetic tools also appears among stock agents and this would seem to be crucial among those intermediaries who do not believe in these technologies and, therefore, would not have the motivation to learn.

Producers have a variety of attitudes and perceptions regarding genetic selection tools. For example, Ooi et al. [13] and Jakku et al. [6] mention the challenge that producers face in making decisions regarding the adoption of new technologies. Even though participants recognised that genetic tools are a worthwhile technology, their adoption has been affected their reliance on traditional practices. This includes visual selection of animals on structure, temperament, and appearance (phenotype). For example, one participant said “you just go you just go back to the old school of how we all bought bulls for years and years and years, […] like it’s technology’s there, it’s a great technology, but you still got to use the old school a bit” (ID10). For some participants there was a disconnect between genetic information and the appearance of the bull. Similarly to claim by Morris and Holloway [31], who identified that problems arise when the desired ideal animal (a combination of genetics and phenotype) is in practice difficult to identify. For instance, from the perspective of a commercial beef producer, “… and their whole program is just reliant on figures, figures, figures, figures, and there’s no way in hell, I would buy a bull off [them] but I mean, they have impressive EBVs” (ID10). For a minority, there was a level of risk with genetic tools; for example, one participant said “It scares some breeders. Yeah, they don’t want to open up the closet to see how they’re tracking, you know, they might be doing well just looking [at the animal], wise and be seen as successful. But yeah, that’s probably some of the reasons why breeders don’t adopt that [genetic tools]” (ID2).

Additionally, specific technical aspects such as data accuracy, on-farm data collection, and lack of results are also suggested challenges that reduce adoption. The term accuracy, associated with each EBV of genetic evaluations, indicates the volume of data incorporated in the calculation of the respective EBV. A high precision value implies a greater probability that the EBV reflects the genuine genetic merit of the animal and, at the same time, is more stable because additional data related to the animal, its offspring or its relatives has a small effect [39]. Sloane and Walker [37] published that the main factor that leads to the negative perception of BREEDPLAN (2024) is related to the accuracy of the information. The relationship between the animal’s genetic data and the expected performance in production systems emerges as a barrier to adoption. For some participants the selection of bulls exclusively using genetic data has not resulted in improvements in the performance on farm, accentuating the negative perception of the use of EBVs. For example, one producer said “… we want some females from a well-known. And now they’re in the top five or seven 600 days worth and all these type of things. But when we get them home, they’re not suited to our country (farm) so that their genetic potential might be there … but they don’t live up to their potential in our country (farm)” (ID6). Additionally, those participants who used the term ‘accuracy’ were referring to the precision and reliability with which phenotypic data is collected. For example, one participant said “… that’s makes pretty hard to use accurately EBV, and there’s a lot of people […] are breeding cattle in big areas, like where a lot of people that would not be able to get those birthdays, and they just have to be estimated. That is the biggest problem I see currently with the EBV system” (ID13).

Data collection and recording was another barrier, particularly in relation to recording phenotype traits practicality. This is consistent with what was published by Bell and Sangster [4] who express that the environment from northern Australia creates a difficult condition in which to collect data consistently. One participant said “the biggest problem that I see is birth dates. A lot of that data replot relies on birth dates, the actual getting a correct birth date of an animal, and in Queensland and the bigger areas, ours, for instance, it’s virtually impossible to get that except for a small amount of cattle” (ID13). This was reiterated by another participant, “birth weight probably is one that it’s probably not practical for most” (ID12). Birth date is important in genetic evaluation programs as it is usually adjusted for covariates such as date and weight at birth. Therefore, not having accurate data at birth also affects other traits of economic importance such as growth traits (200–400–600 day weight) and days to calving. Another aspect is related to the continuity of the collection of data, required to maintain an updated base population in EBV genomics estimates. For instance, “… we still need someone to be recording the phenotypes so that you can continually keep your accuracies up and record the genetic predictions” (ID1). Another barrier is the lack of economic incentive towards those who collect phenotypic data and hair samples for genomic analysis. Some participants remarked regarding collection data that “time and money, and probably no incentive, you need to work, we’re the ones doing all the hard work on the ground to collect this raw data. And there’s quite no incentive there … it’d be nice to be rewarded somehow through. Yeah, financial, yeah, some sort of incentive there to keep collecting the data” and “I think probably the effort to collect it and the costs, I don’t think they see that that justifies” (ID2).

#### 3.1.4. Generational Attitudes

Age was a factor of adoption in the northern Australian beef industry. One participant expressed “I certainly think probably younger people in the industry, who are maybe more accepting of it … be more willing to uptake” (ID7). Additionally, another said “I think going forward as the younger generation sort of take over a lot more, I think you’re going to find going forward, it’s going to be probably something that’s going to be used pretty much 80% of the time, if not more” (ID13). However, there was a change in trend for older generations, because “there’s still some in the older generation that are looking at it and trying to do it and be a part of it” (ID12). Staying relevant in the beef industry could be a driving motivation for older generations. The consistency that age is a crucial factor in the adoption of genetic tools mentioned by the participants in this study seems to contrast with what was found in the review of other studies. In several investigations, age has been demonstrated as a factor that affects adoption processes, although there was inconsistency in the results found [35,36]. For instance, Martin-Collado, Díaz [40] found that age was not found to generally affect producers’ attitudes towards breeding in all breeds analysed; however, it was significant for certain breeds and breeding systems. Even more, Sitienei, Gillespie, and Scaglia [41] express that producers age has shown mixed effects reliant on beef management practices.

### 3.2. Genetic Tools and Traits Preferences

To answer RQ2: What are the preferred genetic tools and why? Stakeholders were consulted about the use and preferences of genetics tools in their enterprises.

#### 3.2.1. Genetic Tools Preferences

Through the interview process, participants expressed what genetic tools support the decision-making process in their beef production systems. Among those participants who use genetic tools, estimated breeding values (EBVs), the use of genomics (gEBVs) and polled gene testing were mentioned. Nevertheless, levels of acceptance would appear to differ among stakeholders. For example, stakeholders recognise the noticeable adoption of polled genetics among producers. One participant expressed “I think there’s a lot more uptake and acceptance, or certainly a lot more acceptance of polled […] there’s been a real growth in the demand for polled bulls in sales probably in the last five years” and “I think there are certain things within genetics that people have accepted quite readily. So for example, the gene poll testing, and that’s something very definite, then people can see a result” (ID7). This could be explained by advances in the accuracy of genetic tests and the usefulness of the selected trait to production systems in financial, operational, and animal welfare terms [42]. Differentially, the acceptance of EBV is beyond technical aspects such as “gains in science into our businesses and production systems” (ID7) among some participants and on marketing aspects by others such as “I often say like EBV is used as a marketing tool, not as a breeding too” (ID6). The results of the interviews suggest that the preference for the genetic tools used is related to optimise the market suitability of the animal. For instance, one participant said “y so genetic tools, whether that be, you know, EBV or DNA testing [genomics], or the combination of both, is really important to or harnessing the science that we have available is really important to push forward the performance of those cattle, the suitability of them to the environment, which again, is going to return you more product per hectare product for whatever your cost base is” (ID8).

#### 3.2.2. Traits Sought by Beef Producers

The genetic traits sought in beef cattle are influenced by production systems in northern Australia and also by the preferences and characteristics of the producers. More traditional producers focus their attention on visual aspects of the animal, such as structure and length of the animal, and behavioural aspects, such as temperament. One participant clearly expresses the differences in the desired temperament of the animal with respect to the size of the production systems: “temperament is one thing on big properties it’s not looked at as much, because the bigger the herd, the less they actually see the individuals trying to chase them. The smaller the herd, the more opportunities, the beast see has have the opportunity to see the person chasing them. So temperament is huge” (ID10).

Regarding the traits sought with genetic data, the opinions and priorities among interviewees seem to be diverse. On one hand, some stakeholders look for growth characteristics. because those traits are associated with productivity and the profitability they obtain through the final sale of the animal. Others focus on birth weight to reduce the probability of difficulty during calving sessions. Some participants mention fertility traits because the number of calves achieved in production systems is also a variable that directly affects the productivity and profitability of beef enterprises. According to Sloane and Walker [37] through a national survey, it was found that beef producers seek genetic gains mainly in fertility, temperament and calving ease. Finally, and associated with the animal breed and markets, when the level of fattening is critical, carcass traits would seem to be relevant.

### 3.3. Genetic Information Resources

To answer RQ3: ‘What is the source of information of beef producers for content related to genetic improvement?’ the main sources of information, perceptions about the role of breeding societies and preferences regarding extension activities were analysed.

#### 3.3.1. Looking for Genetic Information

Participants were asked what sources of information they usually use regarding genetic information in northern Australia beef production. Through the interviews, various perspectives were considered by the participants, such as the experience and relevance of who provided the information and information transfer strategies. As a general result, it was found that there is no predominant source of genetic information, but this is probably due to the variety of roles among the stakeholders who participated in this study. Among the participants, there were differing opinions on the availability of genetic information for northern Australia beef producers, “there is not much information out there” (ID14) versus “I’m sure if they’re [producer] looking for it, they can find it” (ID4). Furthermore, some participants were unable to specify any predominant source of information with one participant expressing “It’s hard to say exactly where bull buyers are getting that information from, but you’d say it to be a wide range of, from their neighbour to Facebook to whatever they want to read” (ID11), and also said “it varies I suppose, like, it all depends … that’s the other thing that’s changed hugely in the last three years”.

For commercial producers, the sources of information are stud producers, mainly those who have a relevant background in the production of bulls for sale. One participant said “families that have been breeding animals for years and years and years, there’s a fair chance that they’ve got a great line […] and that does have something to do with the EBVs too, but more so we’re gonna looking [visual selection] at them” (ID10). This highlights that trust in the source of information is crucial for the process of adoption of genetic tools. This aligns with what was reported by Sloane and Walker [37] that the main source of information is peers. Another important source of information frequently mentioned by participants is the summaries of bulls or booksales provided by stud-producers. However, even though the information was available in sales catalogues, it would not necessarily imply that the information was interpreted and used efficiently. For instance, one stud producer said “the majority of people probably think about it when they go for bull sale, and they read the catalogue and they [producers] see EBV, on this animal and then decide the better find out what it means […] they’re just going freak to the figures” (ID13).

#### 3.3.2. The Role of Breed Societies

Across northern Australia, diverse breeds and crossbreds are used in beef production [1]. Beef producers who focus on a particular breed have the possibility of getting to know each other through a breed society. The breed societies aim to promote the use of a breed and improve its genetics so that it is adapted to the production conditions and market demands. Participants were asked about their perception of the role that breed societies play in the dissemination of genetic tools among their members to increase their use and promote genetic improvement. The results of the interviews suggest that there are differences between breed societies in terms of the role played in encouraging the adoption of genetic tools. Martin-Collado, Díaz [40] shows that the breed raised by producers had an important effect on producers’ attitudes to breeding tools. In our study, for instance, one participant said “It’s certainly different between breeds, there are some breeds that are still fairly reluctant on data recording” (ID3). Additionally, breed societies would have to deal with differing opinions amongst their members regarding the usefulness of genetic tools such as EBV and genomics. A participant expressed it clearly, “I think they should have a big, big role and a big role in. Firstly, there’s like in the beef industry, at the moment, there’s a lot of breeders to say: -oh, no, I’m never going to do that-, even some of the younger ones, then there are other the other breeders who are going into it full on” (ID13). The interviews suggest that some breed societies and their members have an active role in the use of genetic tools in northern Australia and are driving the trend of increasing the use of genetic tools.

#### 3.3.3. Extension Methods

Extension refers to the use of educational and communication strategies aimed at empowering beef producers to recognise potential improvements in decision-making [42,43]. Participants were asked about their preferences regarding participating in extension activities with most highlighting their preference for activities with a small group of producers and face-to-face. For instance, one participant said, “I think it’s definitely got to be face to face in small groups, whether that’s in a workshop setting, or whether it’s on-farm field days or the likes” (ID8), and another said, “If you’re doing like a bigger overall presentation, then you can break into smaller groups, which I think you do in in small groups to get people to ask question” (ID3). This aligns with what was published by Nettle et al. [44] where the authors through an exhaustive review of agricultural extension activities across Australia, conclude that small-group learning and direct coaching methods were most effective. The importance of break time during extension activities was also highlighted as this is where producers usually approach each other or the presenters to talk, ask questions or seek clarification. Expressions like “people are too scared to speak up” (ID3) or “some people don’t like to ask questions on the floor [on the field in front of other producers], but they all privately do it” (ID4) were mentioned by two participants.

Regarding the location of extension activities, participants expressed a clear preference for on-farm activities such as field days and workshops. One participant said “field days and seeing … that means to be keeping these results, and for them to be talking to people that are already doing it” (ID5). However, one person said an “information day on a property I think works the best where you got speakers, you there’s people come together, not always easy to do, especially in the north with how spread out people are” (ID2), recognising the difficulty to do this in northern Australian. Remoteness is a factor to consider in adoption processes in northern Australia [4]. This aligns with Nettle et al. [44], who suggests that researchers could participate in digital platforms to improve and collaborate with the extension agents to develop content crucial to the producer’s needs.” The timing of the extension activity and who the speaker is also seems to be a crucial issue, “if it’s a course or a one day workshop, or those sorts of things, I think anything more than one or two days is really hard to get people to go to, because of the restraints on their time and their business” (ID7) one participant said. The expertise of the person who presents the knowledge was highlighted by several participants. For example, one participant said “learning from someone with their skin in the game. So what I mean by that is, is a person that’s you know, rely on the EBVs to make money” (ID10), and another said “we think that I would trust other graziers… if they went to someone’s place, and they explained what they’re doing and why. And these are the benefits and things like that, I think that probably has the biggest impact, because they can relate to it, and they can they can see it” (ID7).

## 4. Theoretical Framework

The theoretical multi-level framework UTAUT/UTAUT2 [7] was considered in this study. The decision to adopt genetic tools in northern Australian beef production systems appears to be affected by various contextual attributes, both for higher-level and individual-level contextual factors. In a personal decision-making model, a new approach emerges when there is a gap between the capabilities of current technology and the demands imposed by evolving agricultural circumstances [45].

Considering the baseline model, habits, and individual beliefs affect behavioural intention [7,17] and the results of the interviews in this study suggest that age is a factor that affects decision-making regarding the adoption of genetic tools. Age is one of the four (age, gender, experience and voluntariness of use) attributes that can influence the factors (performance expectancy, effort expectancy, social influence and facilitating conditions) that affect the intention and behaviour related to the use of technology [7]. The results of how age effect technology adoption in agriculture are divergent [36,46,47]. It is common to find reports from the beef industry from northern Australia with demographic data from beef producers where the most common age is between 55 and 65 years [37,48]. Communication strategies to increase the adoption of genetic tools among beef producers from northern Australia should also consider the characteristics of the age groups. Although those interviewed mention a change in trend regarding the use of social media networks, communication focused on in-person relationships is still essential.

In relation to the high-level contextual factors, the environmental attributes are about the physical context and conditions in which the technology is used [7,49]. The conditions of northern Australia beef production, such as temperatures, periods of drought and flooding, large areas of land, remote areas, low stocking rates, limited connectivity and targeting production for the live export market, have influenced the search for beef cattle that are adapted to the environment [1,4]. From the interviews, it identified that there is currently a process of change occurring in adoption of genetic tools under these production and environmental conditions. Where the differentiation of animals through their genetics begins to be relevant in the beef industry, whether as a marketing strategy, profitability or productivity, even the stakeholders who expressed scepticism recognise the rise in genetic tools influence on the northern Australian beef industry. Specific technical aspects such as on-farm data collection related to the environmental attributes and lack of results or data accuracy related to technology attributes are also identified issues. The organisational attributes refer to the social context of the technology acceptance [7]. In the context of this research, the adoption of genetic tools would seem to be led by large beef enterprises. Partnerships between stud producers seem to be an effective strategy to promote the use of genetic tools among their clients and obtaining the financial reward for the time, effort and costs addressed for the genetic evaluations they offer to their clients encourage adoption processes. From the participants, it is evident that there are different perceptions about the role that breed societies have or should have to contribute to motivating the use of genetic tools among beef producers in northern Australia.

Finally, considering the individual-level contextual factors, experience and education could be considered user attributes and play a crucial role in technology adoption. The effects of user attributes are one of the individual-level contextual factors of the multilevel framework of technology acceptance and use [7]. Consequently, the level of understanding and education might influence the acceptance and use of a technological tool through individual beliefs. Individuals’ perception of themselves as ‘traditional’ affects their willingness to consider new resources, such as the use of genetic tools in the decision-making process. However, through the interviews, a change from even traditional producers towards a recognition of new technologies is emerging.

## 5. Limitations of This Study

This study has several limitations that should be acknowledged. Although purposive sample is appropriate for qualitative research, this could introduce a selection sample bias by stakeholders who decide to participate because they are already engaged in genetic and extension activities in the beef industry from northern Australia. Additionally, the findings may be influenced by participants’ self-reported perceptions, which could introduce personal bias. While the sample size is adequate for exploratory and qualitative analysis aimed at generating deeper insights, it may not represent the diversity of roles, regions, breeds and market channels within northern Australian beef industry. Another limitation is the partial alignment between the codes generated inductively and the theoretical framework. These limitations should be considered when interpreting the results of this study. Future research should aim to address these constraints, with more representative samples, mixed-method approaches, and the inclusion of case studies (e.g., exploring economic motivations) to further validate and contextualise the perceptions reported in this study is needed.

## 6. Conclusions

This research sought to explore the motivations, barriers and preferences of beef producers in northern Australia with respect to the adoption of genetic tools from the perspectives of various stakeholders. Interview results suggest that there is low adoption of genetic tools in northern Australia, although there has been a change towards an increase in adoption intentions in recent years. Among the motivations for the adoption of genetic tools were the usefulness of information that cannot be obtained through visual inspection of the animal, but fundamentally if there is an economic benefit. Among the barriers that limit adoption, the lack of accuracy and not observing results (productively and economically) in production systems accentuates the attitude of resistance to change, supporting traditional practices of visual selection of animals. Here the differences arise in terms of the scale of production, the decision-making processes in large companies focus more on genetic information as validation of the assumed costs. Understanding genetic tools also emerges as a great barrier to adoption. The difficulty of data collection in northern Australian conditions is also a limiting factor in achieving reliable genetic values. Regarding extension activities, most participants highlighted their preference to participate in activities with a small group of producers and face-to-face delivered by beef producers with expertise. The deeper understanding gained through the interviews conducted and analysed in this study could be useful in the design of future quantitative surveys. Consequently, future research should include a larger scale survey to gain insight into a representative sample of northern Australian beef producers, and then this information together with interview data could be used to design an extension activity to promote the use of genetic tools.

## Data Availability

All data used in the current study are confidential according to CQUniversity Australia Human Research Ethics Committee procedure.
